# Research on Vibration Accumulation Self-Powered Downhole Sensor Based on Triboelectric Nanogenerators

**DOI:** 10.3390/mi15040548

**Published:** 2024-04-19

**Authors:** Rui Wang, Jianchao Ren, Weibo Ding, Maofu Liu, Guangzhi Pan, Chuan Wu

**Affiliations:** 1 Shaanxi Shaanxi Coal Caojiatan Mining Co., Ltd., Yulin 719100, China; wr2024@yeah.net (R.W.); renjianc888@163.com (J.R.); dingweibo999@163.com (W.D.); liumaof@163.com (M.L.); 2 Faculty of Mechanical and Electronic Information, China University of Geosciences (Wuhan), Wuhan 430074, China; panguangz@126.com

**Keywords:** triboelectric nanogenerator, self-powered, vibration sensor, high output performance, vibration accumulation

## Abstract

In drilling operations, measuring vibration parameters is crucial for enhancing drilling efficiency and ensuring safety. Nevertheless, the conventional vibration measurement sensor significantly extends the drilling cycle due to its dependence on an external power source. Therefore, we propose a vibration-accumulation-type self-powered sensor in this research, aiming to address these needs. By leveraging vibration accumulation and electromagnetic power generation to accelerate charging, the sensor’s output performance is enhanced through a complementary charging mode. The experimental results regarding sensing performance demonstrate that the sensor possesses a measurement range spanning from 0 to 11 Hz, with a linearity of 3.2% and a sensitivity of 1.032. Additionally, it exhibits a maximum average measurement error of less than 4%. The experimental results of output performance measurement indicate that the sensor unit and generator set exhibit a maximum output power of 0.258 μW and 25.5 mW, respectively, and eight LED lights can be lit at the same time. When the sensor unit and power generation unit output together, the maximum output power of the sensor is also 25.5 mW. Furthermore, we conducted tests on the sensor’s output signal in conditions of high temperature and humidity, confirming its continued functionality in such environments. This sensor not only achieves self-powered sensing capabilities, addressing the power supply challenges faced by traditional downhole sensors, but also integrates energy accumulation with electromagnetic power generation to enhance its output performance. This innovation enables the sensor to harness downhole vibration energy for powering other micro-power devices, showcasing promising application prospects.

## 1. Introduction

**Figure 1:**
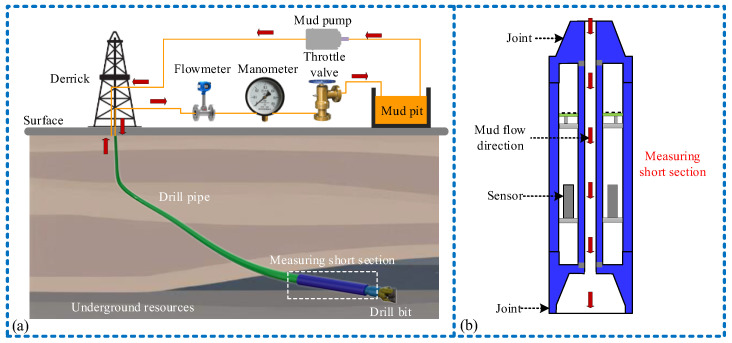
Drilling and the installation method of the sensor: (**a**) the drilling schematic diagram; (**b**) schematic diagram of sensor installation.

Drilling is a technique that involves using drill rigs or other equipment to bore holes in the ground or underwater to gather information about underground resources and assess their potential for extraction [1,2], as illustrated in Figure 1. However, during the drilling process, the interaction between the drill bit and the formation or between the drill string and the wellbore wall can result in vibrations of the drilling equipment [3]. Excessive vibration and its severe impacts can cause the wear, deformation, or complete failure of various components within the drilling tool and may even result in tool breakage, leading to serious wellbore accidents and jeopardizing drilling safety [4]. Therefore, it is necessary to measure the vibration information of the drilling tool in real time. Currently, there is extensive research on downhole vibrations, which has yielded numerous results. Some scholars have conducted extensive research on the transmission laws and models of drill string vibration [5,6]. Some scholars utilize acceleration sensors [7,8], gyroscopes [9], and strain gauges [10] to measure downhole vibration.

However, the traditional vibration measurement methods mentioned above all have some limitations when applied in drilling conditions. If sensors are installed on the surface, it can lead to deviations between the measured results and the actual downhole vibration parameters, failing to reflect the real situation; if sensors are installed at the bottom of the well, it can bring a series of power supply issues [11,12]. The current commonly used power supply methods for downhole equipment mainly consist of external cable and battery pack supply. If external cables are used, they need to be threaded through the entire drill string, significantly reducing construction efficiency and substantially increasing costs. If battery packs are used for power supply, the drill string needs to be fully raised to the surface and new battery packs replaced once their charge is depleted. This process typically takes several days, consequently reducing drilling efficiency. Therefore, sensors with self-power supply capability are undoubtedly more suitable for downhole work conditions.

As drilling technology advances and electronic equipment undergoes extensive miniaturization, the concept of micro-nano energy and self-power has emerged. The triboelectric nanogenerator (TENG) serves as a notable example. Through ongoing exploration by researchers, significant progress has been achieved in the self-powered sensing and energy harvesting fields [13]. Regarding self-powered sensing, TENG has been successfully employed in monitoring acceleration [14,15], angle [16,17,18], displacement [19,20,21], and wearable device sensors [22]. Simultaneously, significant research progress has been achieved in the parameter measurement of various types of vibration sources [23,24,25]. In the realm of energy harvesting, TENGs demonstrate the capability to gather and reuse diverse forms of energy, including ocean energy [26,27,28,29], wind energy [30,31], body movement energy [32,33,34], environmental mechanical energy [35], and rotation energy [36]. This highlights the substantial advantages of using a TENG as a sensor with energy collection functionality. Furthermore, several scholars have integrated TENGs into the domain of downhole sensors, enabling the monitoring of drilling tool vibration [37], rotational speed [38], and other pertinent parameters. In this research, we propose a self-powered vibration sensor with high output performance tailored for downhole working conditions. This is achieved by integrating the TENG generation principle with downhole vibration measurement technology. In comparison to traditional downhole vibration measuring devices, this sensor not only enables self-powered vibration measurement but also harnesses vibration energy to provide power to other downhole modules. This capability is anticipated to address the power supply challenges encountered in downhole equipment, consequently enhancing drilling efficiency. Such advancements offer a reference for the advancement and utilization of self-powered sensors within downhole drilling tools.

## 2. Structure and Working Principle

### 2.1. Structural Design

**Figure 2:**
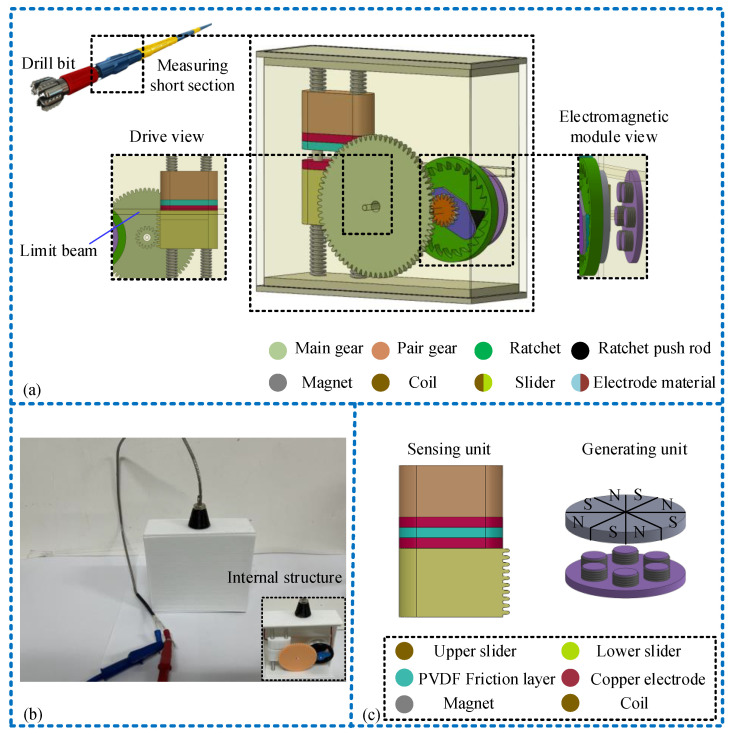
Sensor structure design and model diagram: (**a**) sensor 3D model diagram and detail display; (**b**) physical image and profile of sensor; (**c**) two-dimensional models of different elements of the sensor.

The drill tool comprises a drill bit, drill collars, and a drill pipe. Vibration intensifies the closer one moves toward the drill bit. Hence, the vibration accumulation sensor is installed within the measurement short section near the drill bit position, as shown in Figure 1b and Figure 2. As depicted in Figure 2a, the sensor is cuboid-shaped and vertically installed within the drilling tool sub, comprising both a sensing unit (TENG) and a power generation unit (EMG). The sensing unit comprises a set of sliders, two copper electrodes, and a polyvinylidene difluoride (PVDF) friction layer film sandwiched between copper electrodes. The PVDF film has a thickness of 0.1 mm, while the copper electrode on the slider’s contact surface measures 0.05 mm in thickness. The power generation unit comprises an electromagnetic module connected via gear and ratchet transmission. A rack is situated on the side of the sliding block in the sensing device, forming rack transmission with the pinion. The pinion and the large gear drive the ratchet wheel to rotate, enabling it to accelerate the rotation of the external gear in the same direction. Behind the external gear, the interval magnet (considering actual working conditions and potential de-Gaussing effects, EH NdFeB magnets are chosen) and a copper coil are installed. Moreover, a limit beam is installed on the side of shell to separate the upper and lower slide blocks during vibration, enabling the measurement of vibration parameters. Figure 2b displays the physical picture and internal structure of the sensor, while Figure 2c illustrates a two-dimensional model diagram of the sensor’s sensing and power generation units. The mechanical transmission settings within the sensor are detailed in Table 1.

### 2.2. Working Principle

**Figure 3:**
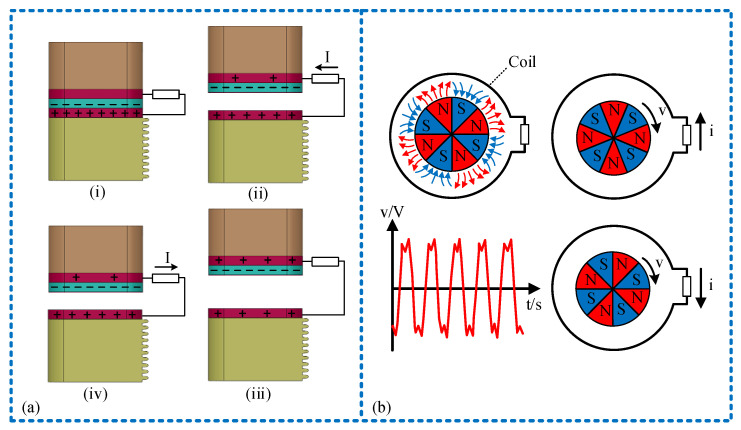
Schematic diagram of sensor working principle: (**a**) charge transfer schematic diagram of sensing unit, among them, (i): contact between the PVDF friction layer and the lower copper electrode begins; (ii): separation starts between the PVDF friction layer and the lower copper electrode; (iii): the upper slider reaches its peak position; (iv): approach begins between the PVDF friction layer and the lower copper electrode; (**b**) working principle diagram of power generation unit.

The working principle of the sensor is depicted in Figure 3. In the sensing unit, the PVDF friction layer and copper electrode establish contact to work in a vertical contact separation mode. Initially, the PVDF friction layer and the lower copper electrode are in contact, as illustrated in Figure 3a(i), due to the electronegative surface inducing different charges in equal amounts. When external vibration excitation is applied, the lower sliding block remains fixed due to the influence of the limit beam. The upper slider ascends, causing the PVDF friction layer to separate from the lower copper electrode. This separation led to the transfer of positive charge from the lower copper electrode to the upper copper electrode, resulting in the formation of a displacement current and an increase in potential difference, as depicted in Figure 3a(ii). As the upper slider reached its peak position, the potential difference reached its maximum, as illustrated in Figure 3a(iii), Following this, as the upper slider descends, and as the distance between the PVDF friction layer and the copper electrode diminishes, a positive charge flows from the upper copper electrode to the lower copper electrode, initiating a decrease in potential difference, as shown in Figure 3a(iv). Ultimately, the upper slider reverts to its initial position, causing the potential difference to decrease to zero. Consequently, within a vibration cycle, the sensing unit generates a pulse wave, facilitating the measurement of vibration frequency through the frequency of the collected signal waveform.

In the power generation unit, reciprocating vibrations are transformed into a clockwise circular motion by the mechanical structure, propelling the magnet rotation via the external gear. Within the closed loop, as the coil and magnet rotate relative to each other, cutting across the magnetic induction lines, an induced current is generated. The direction of this current can be determined using the right-hand rule. Using a group of coils as an example, as depicted in Figure 3b, the induced current direction can be determined by the right-hand rule. When the magnet’s N pole is positioned above the coil, the induced current is counterclockwise, and when the magnet’s S pole is above the coil, it is clockwise. The sensor’s vibration excitation can be transformed into a clockwise circular motion, realizing the accumulation of vibration. Consequently, the power generation unit produces a continuously changing alternating current output signal.

**Figure 4:**
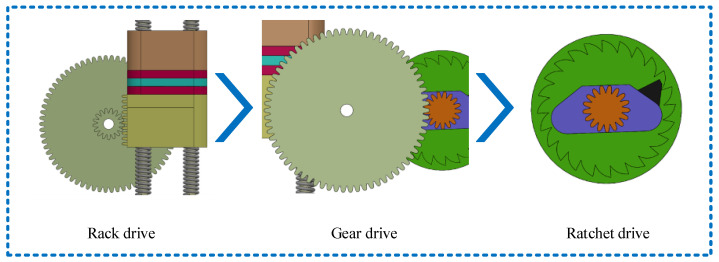
Analysis of the process of transforming vibration form into circular motion.

Furthermore, a detailed explanation of the transformation of vibration into circular motion is provided. As depicted in Figure 4, when the upper slide block and the lower slide block make contact, both descend due to inertia and gravity. The side rack of the lower slide block engages with the pinion, causing the pinion assembly to rotate. Subsequently, the large gear drives the ratchet to rotate, and a sliding member is installed at one end of the ratchet teeth. The external gear is rotated in a constant direction to realize the conversion and accumulation of vibration energy.

## 3. Tests and Results

### 3.1. Experimental Facilities

**Figure 5:**
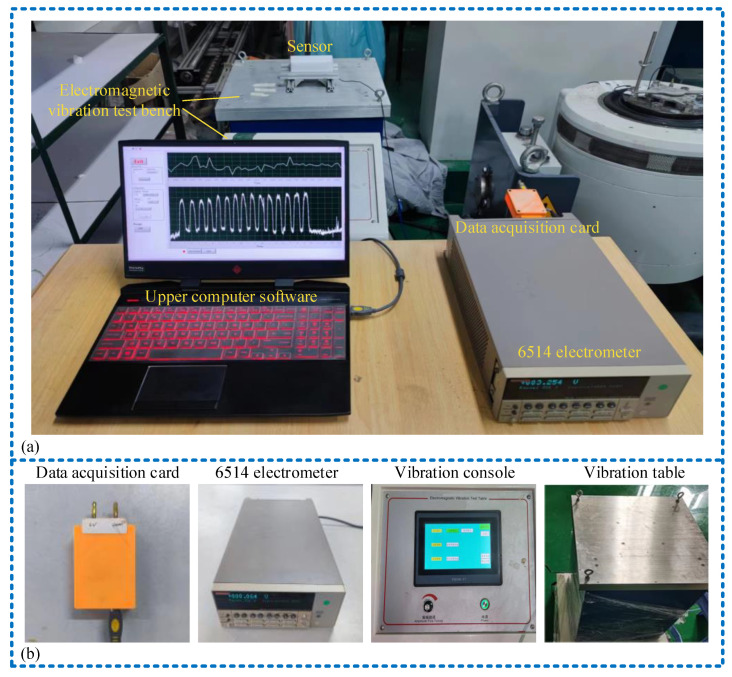
Experimental facilities: (**a**) a picture of the experimental facilities; (**b**) some details of the experimental facilities.

To assess the sensing and power generation capabilities of the sensor, we constructed a vibration simulation apparatus mimicking downhole drilling tool vibrations, as shown in Figure 5. Positioned at the vibration platform, the sensor detects vibrations generated at various frequencies by adjusting the controller. The sensor then generates a voltage signal, which is relayed through a data acquisition card (USB5632, ART Technology Co., Ltd., Beijing, China). Subsequently, a 6514 electrometer (Keithley Inc., Cleveland, OH, USA, 6514) collects the data, which are transmitted to PC software for displaying the signal waveform.

### 3.2. Research on Sensing Performance

**Figure 6:**
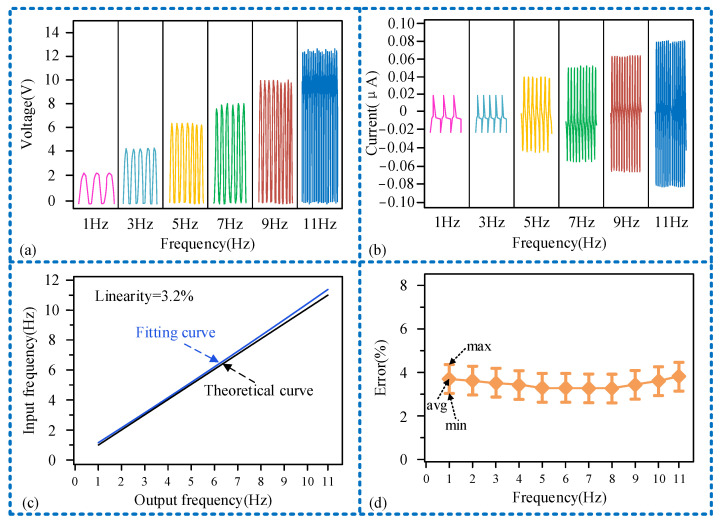
Research results of sensor sensing performance: (**a**) waveform of output voltage varying with frequency; (**b**) waveform of current varying with frequency; (**c**) sensor linear correlation graph; (**d**) sensor measurement error trend chart.

Firstly, we researched the capability for measuring the vibration frequency of this sensor, assessing its sensing performance in this regard. Illustrated in Figure 6, we investigate the variations in output voltage and current across various vibration frequencies detected by the sensor. As the frequency escalates, both the output voltage and current exhibit an uptrend. At the peak frequency of 11 Hz, the output voltage registers at 12.3 V, with a corresponding current of 0.078 μA, as depicted in Figure 6a,b, respectively. Obviously, the frequency measurement range of the sensor has been determined to be from 0 to 11 Hz. Since the vibrations in geological drilling are primarily caused by the collision or friction between the drill string and the rock during rotation, typically around 2 Hz, this measurement range meets the practical requirements of downhole vibration measurement sensors. When the external vibration excitation reaches 11 Hz, the output voltage and power peak; thereafter, as the external vibration frequency continues to increase, the sensor’s output signal waveform gradually becomes distorted. Considering that the sensing unit functions as a symmetrical double-spring vibration system, the upper and lower slide blocks are separated by a limiting beam. Frequency measurement is achieved through contact separation between the PVDF friction layer on the interface of the two components and the copper electrodes. To demonstrate the correlation between the measured frequency and input frequency, theoretical and actual curves of both were plotted. As shown in Figure 6c, after conducting multiple tests on the sensor’s output frequency at different input frequencies, the measurement results were analyzed using the least squares method to obtain a fitting curve. The test results indicate that the linearity of the sensor is 3.2%, and its sensitivity is 1.032. Following this, an analysis of the sensor’s measurement error was conducted. Numerous tests were performed on the sensor’s output signal frequency at various frequencies to establish its error range. The curve was plotted by averaging the maximum and minimum errors obtained from these tests using the arithmetic mean method. It was observed that the sensor’s measurement error initially decreased before increased, with the maximum average measurement error being less than 4%, as illustrated in Figure 6d. These results show the sensor’s relatively dependable sensing performance.

### 3.3. Research on Power Generation Performance

**Figure 7:**
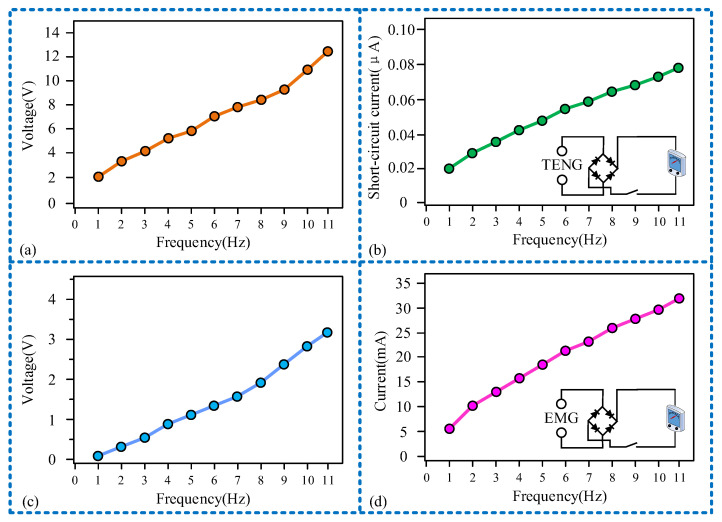
Sensor output voltage and current change curve with frequency: (**a**) the relationship between the output voltage and frequency of the sensor unit; (**b**) the relation curve of the short-circuit current and frequency of the sensing unit; (**c**) the relationship between the output voltage and frequency of a generating unit; (**d**) the relationship between the short-circuit current and frequency of a generating unit.

After exploring the sensing performance of the sensor, we proceeded to investigate its output power performance. The sensor’s output power encompasses the power of both the sensing unit and the power generation unit, as illustrated in Figure 7. Initially, we examined the relationship between the output voltage and current of the two units with frequency variation. It is observed that, at the maximum range of 11 Hz, the output voltage of the sensing unit is 12.3 V, with a short-circuit current of 0.078 μA, as shown in Figure 7a,b. The output voltage of the power generation unit is 3.2 V, with a current of 32 mA, as displayed in Figure 7c,d. Additionally, Figure 7b,d present the rectifier circuits for the TENG and EMG, respectively. The output voltage and current of both the sensor unit and the generator unit increase as the frequency rises. Consequently, the maximum range frequency (11 Hz) has been chosen for the matching of the output power of the sensor and load in subsequent studies.

**Figure 8:**
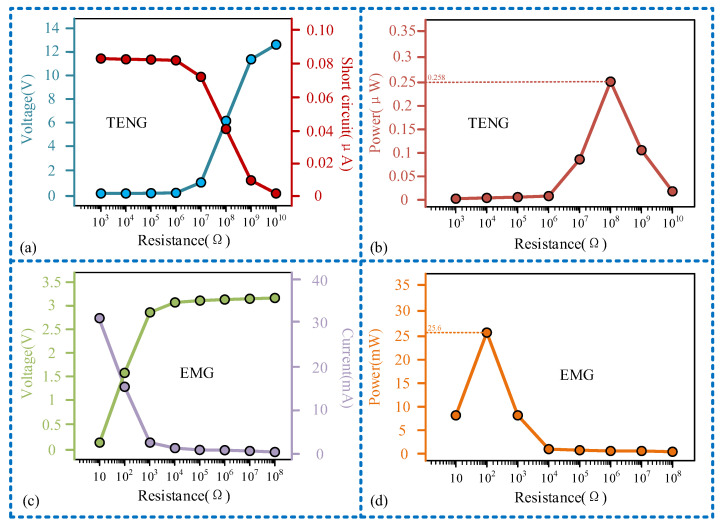
Research results of the output power performance of the sensor: (**a**) the relationship between the output voltage and current of the sensing unit and the external load; (**b**) the relationship between the output power of the sensor unit and the external load; (**c**) the relationship between the output voltage and current of a generating unit and the external load; (**d**) the relationship between the output power of a generating unit and the external load.

**Figure 9:**
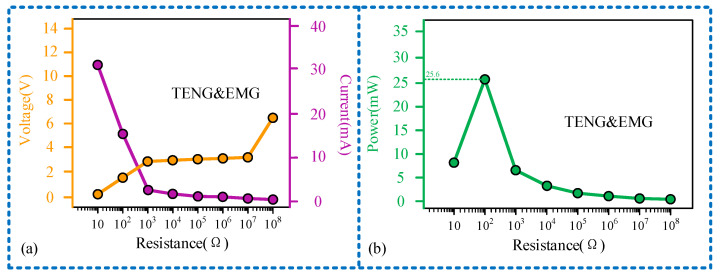
Experimental results of TENG and EMG hybrid output: (**a**) the relationship between the output voltage and current of the TENG&EMG and the external load; (**b**) the relationship between the output power of the TENG&EMG and the external load.

Load matching experiments were conducted for each unit of the sensor at a frequency of 11 Hz, as illustrated in Figure 8. The relationship curve between the output voltage of the sensing unit and the external load exhibits three distinct stages: gradual decline, rapid decline, and gradual decline once more. Similarly, the relationship curve between the current and the external load displays stages of gradual increase, sharp increase, and gradual increase, as depicted in Figure 8a. Due to the sensing unit operating on the TENG principle, characterized by high voltage and low current, there is a considerable disparity in the magnitudes of output voltage and current. When the external load matches the internal resistance of the sensing unit at 10^8^ Ω, the maximum output power of 0.258 μW is achieved, as depicted in Figure 8b. Conversely, the power generation unit, operating on the principle of electromagnetic induction, exhibits characteristics of low voltage and high current compared to the TENG, as illustrated in Figure 8c. Therefore, the maximum output power is attained with a smaller external load. As demonstrated in Figure 8d, the power output of the generating unit peaks at 25.5 mW, when the external load is 100 Ω. Furthermore, we connected the sensing unit (TENG) and power generation unit (EMG) of the sensor in parallel and studied their mixed output characteristics at a frequency of 11 Hz, with experimental results shown in Figure 9. It can be observed that the mixed output current of TENG and EMG remains largely consistent with the independent output current of EMG, while the mixed output voltage experiences a sudden increase when the load resistance is 10^8^ Ω. This is because the magnitude of the output current from the EMG is much greater than that from the TENG; thus, the magnitude of the mixed output current is close to the independent output current of the EMG. When the external load reaches 10^8^ Ω, the voltage of the TENG increases to over 6 V, increasing the mixed output voltage as well. From the perspective of output power, the maximum value of the mixed output power of TENG and EMG is also nearly identical to the peak value of the independent output power of EMG, with both being 25.5 mW. This also indicates that in the mixed output mode, the power generation performance of the sensor is not significantly affected.

**Figure 10:**
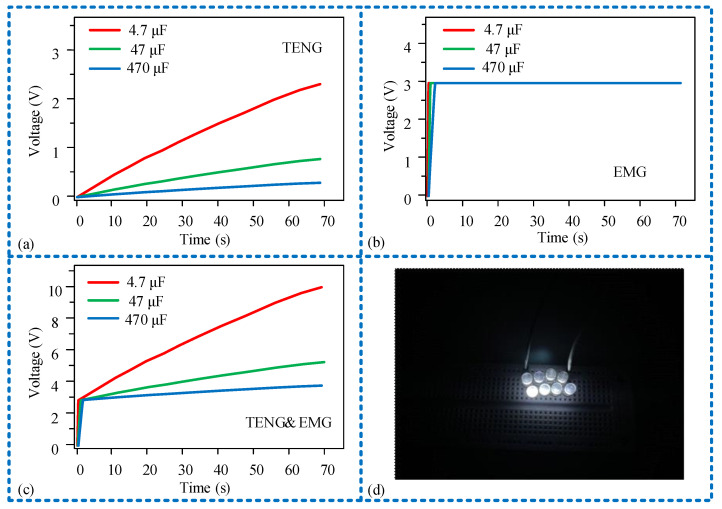
The sensor charges different load capacitors: (**a**) the sensing unit charge capacitors of 4.7 μF, 47 μF, and 470 μF; (**b**) the power generation unit charge capacitors of 4.7 μF, 47 μF, and 470 μF; (**c**) both the sensing and power generation units charge capacitors of 4.7 μF, 47 μF, and 470 μF; (**d**) the sensor lights up 8 LED lights.

After obtaining the output power curves of each unit of the sensor, we conducted further experiments on the sensor’s output power. We connected the sensing unit and the power generation unit of the sensor separately to different capacitors and analyzed the output power performance through the charging curves of the capacitor. Firstly, we individually used the sensing unit to charge capacitors of 4.7 μF, 47 μF, and 470 μF. At 70 s, they were charged to 2.4 V, 0.74 V, and 0.24 V, respectively. It can be observed that the larger the capacitor, the slower the charging rate, as shown in Figure 10a. Next, the power generation unit was employed to charge the capacitors. The 4.7 μF capacitor was fully charged in 7 ms, the 47 μF capacitor in 105 ms, and the 470 μF capacitor in 2.1 s. The voltage across all capacitors reached 3 V. The charging rate of the power generation unit was overall faster than that of the sensing unit, as illustrated in Figure 10b. Finally, integrating the outputs of the sensing unit and the power generation unit for capacitor charging, it can be observed that before reaching 3 V, the power generation unit contributes more to capacitor charging. After reaching 3 V, the charging is continued by the sensing unit. This is because the measured voltage is the voltage across the capacitor terminals, and the output voltage of the sensing unit is higher than that of the power generation unit, allowing the capacitor to continue charging. The charging rate is the same as when the sensing unit charges alone, as shown in Figure 10c. Additionally, the sensor, after rectification through the designed circuit, can illuminate 8 LED lights, demonstrating the potential of the sensor to power external devices.

### 3.4. Research on Downhole Influencing Factors

**Figure 11:**
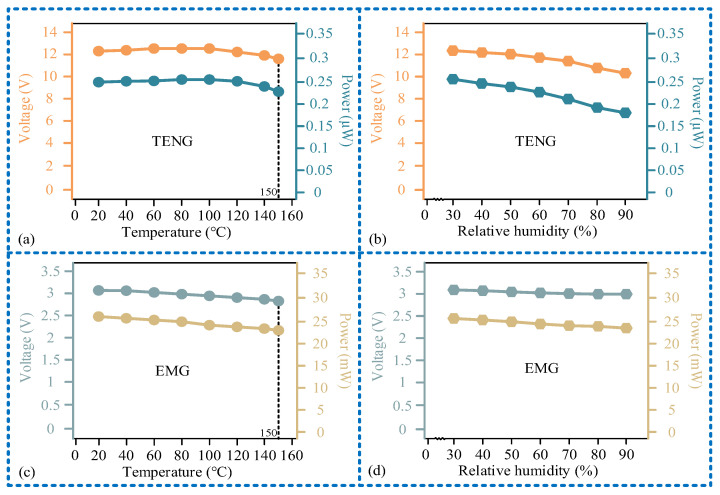
Research results of downhole influencing factors: (**a**) relationship curve of the output voltage, output power, and temperature of the sensing unit; (**b**) relationship curve of the output voltage, output power and relative humidity of the sensing unit; (**c**) relationship curve of the output voltage, output power, and temperature of the power generation unit; (**d**) relationship curve of the output voltage, output power and relative humidity of the power generation unit.

**Figure 12:**
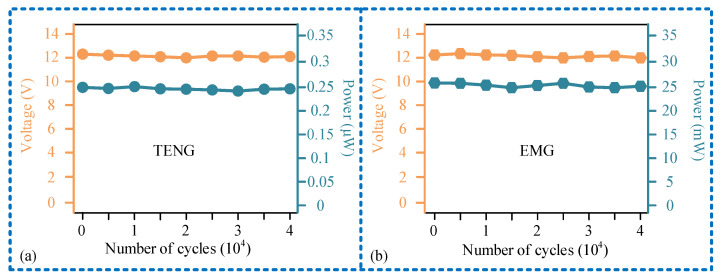
Research results of work stability: (**a**) experimental results of the sensing unit stability; (**b**) experimental results of the power generation unit stability.

Finally, we investigated the impact of downhole environmental factors on the sensor’s output performance, with the results shown in Figure 11. For the sensing unit, as the temperature increases, both its output voltage and output power first increase and then decrease. At a temperature of 150 ℃, the output voltage is 12 V, with an output power of 0.23 μW. As the relative humidity increases, both its output voltage and power decrease. When the relative humidity is 90%, its output voltage is 10.5 V, and the output power is 0.18 μW. The power generation unit is less affected by temperature and relative humidity. It can output a 2.8 V voltage and 23.4 mW real-time power at 150 °C and 30% relative humidity and can output a 3 V voltage and 24.4 mW power at 20 ℃ and 90% relative humidity. In addition, we investigated the working stability of the sensor, as shown in Figure 12. It can be seen that after multiple operating cycles (40,000 times), the output voltage and load power of both the sensing unit and power generation unit hardly change. Considering that the robustness of the sensor is only influenced by temperature and humidity factors, and the attenuation of the output signal under high temperature and humidity conditions is small, the sensor exhibits high robustness.

## 4. Conclusions

In this research, a vibration-accumulation-type self-powered sensor is proposed. It converts reciprocating vibration into circular motion with constant motion direction through special gear transmission and ratchet transmission, thus realizing the transformation and accumulation of vibration energy. The experimental results show that the sensor has a measuring range of 0 to 11 Hz, a linearity of 3.2%, a sensitivity of 1.032, and a frequency measurement error of less than 4%. After rectification, the maximum output power of the sensor unit is 0.258 μW when the external load is 10^8^ Ω. The output power of the power generation unit and TENG&EMG reaches the maximum level of 25.5 mW when the external load is 100 Ω. The sensor has the capability to simultaneously illuminate eight LED lights, thus demonstrating its potential to provide power to micro-power devices. In addition, the experimental results of environmental factors show that the output power of the sensor unit is 0.23 μW at 150 degrees Celsius and 0.18 μW at 90% relative humidity. For the power generation unit, it can output 23.4 mW of real-time power at 150 ℃ and 30% relative humidity and 24.4 mW of power at 20 °C and 90% relative humidity.

Compared to traditional underground vibration measurement devices of the same type, the sensor has two innovative aspects. Firstly, the sensor achieves self-powered vibration frequency measurement by introducing TENG, addressing the issues of low drilling efficiency and significant cost increase associated with the power supply method of traditional downhole vibration measurement sensors. Secondly, the sensor utilizes a vibration-accumulating mechanical mechanism to accumulate power generation, significantly increasing the sensor’s output power and enabling it to potentially power other low-power devices downhole. However, current advancements still have limitations. The sensor’s output signal is both weak and unstable, necessitating external circuit processing before the power supply, which incurs significant energy consumption. To address this, future efforts will focus on refining material surface micro-treatments to enhance output power and stability, thereby maximizing available energy.

## Figures and Tables

**Table 1:** Description of mechanical transmission parameters of the sensor.

Component	Tooth Number	Transmission Ratio (between the Upper and Lower Devices)
Lower slide block rack	12	
		3/2
Pinion	18	
		1/4
Large gear	72	
		4/1
Pawl with gear	18	
		1/1
ratchet	24	

## Data Availability

The original contributions presented in the study are included in the article, further inquiries can be directed to the corresponding author.
